# Deep learning-based multi-omics model to predict nasopharyngeal necrosis of re-irradiation for recurrent nasopharyngeal carcinoma

**DOI:** 10.3389/fonc.2025.1607218

**Published:** 2025-07-15

**Authors:** Xingwang Gao, Yinglin Peng, Shanfu Lu, Yuhan An, Meining Chen, Jun Zhang, Runda Huang, Yiran Wang, Zhenyu Qi, Yao Lu, Chong Zhao, Xiaowu Deng, Jingjing Miao, Yimei Liu

**Affiliations:** ^1^ Department of Radiation Oncology, State Key Laboratory of Oncology in South China, Guangdong Key Laboratory of Nasopharyngeal Carcinoma Diagnosis and Therapy, Guangdong Provincial Clinical Research Center for Cancer, Sun Yat-sen University Cancer Center, Guangzhou, China; ^2^ School of Biomedical Engineering, Sun Yat-sen University, Shenzhen, China; ^3^ Cancer Hospital Chinese Academy of Medical Sciences, ShenZhen Center, Shenzhen, China; ^4^ Perception Vision Medical Technologies Co. Ltd., Guangzhou, China; ^5^ School of Data and Computer Science, Sun Yat-sen University, Guangzhou, China

**Keywords:** recurrent nasopharyngeal carcinoma, radiation therapy, nasopharyngeal necrosis, deep learning, magnetic resonance imaging

## Abstract

**Background and purpose:**

Patients with recurrent nasopharyngeal carcinoma (rNPC) undergoing re-irradiation have a high risk of lethal nasopharyngeal necrosis (NN), which may lead to massive nasopharyngeal hemorrhage or death. Predicting NN is crucial to improve the prognosis of these patients. We aimed to utilize deep learning techniques in combination with multi-sequence magnetic resonance imaging (MRI) radiomics and dosiomics to predict the risk of nasopharyngeal necrosis in patients with recurrent nasopharyngeal carcinoma undergoing re-irradiation therapy.

**Materials and methods:**

117 patients with rNPC were included, comprising pre-treatment multi-sequence MR images (including T1, T1C, and T2 sequences) and a planned re-irradiation therapy dose distribution. A three-dimensional (3D) convolutional neural network (CNN) deep learning network model was utilized to integrate the selected MRI radiomics and dosiomics features. Eight prediction deep learning models were developed for training, 97 cases were used as the training set and 20 as the test set. The performance and prediction accuracy of each deep learning network model were then evaluated.

**Results:**

Thirty-two features correlated with necrosis of rNPC. The model based on multi-sequence MRI radiomics could better predict necrosis. The models combining radiomics and dosiomics features were more accurate for the prediction of NN, especially the model of multi-sequence MRI radiomics plus dosiomics, which showed the best performance in the test set, with an AUC, ACC, and F1-Score of 0.81, 0.75, and 0.74, respectively.

**Conclusion:**

The deep learning model leveraging pre-treatment multi-sequence MRI radiomics and dosiomics of re-irradiation therapy can serve as a potential predictor of NN in patients with recurrent nasopharyngeal carcinoma, thereby improving clinical decision-making processes.

## Introduction

Nasopharyngeal carcinoma (NPC) is a common malignancy affecting the head and neck region (1). Although the implementation of intensity modulated radiation therapy (IMRT) in primary nasopharyngeal carcinoma treatment has improved radiation dose distribution, 10-15% of patients still experience local recurrence (2). Re-irradiation remains the mainstay treatment for patients with recurrent nasopharyngeal carcinoma (rNPC) (3). However, rNPC patients undergoing re-irradiation may develop severe late adverse events, among which irreversible nasopharyngeal necrosis (NN) accounts for the largest proportion (4, 5). Approximately 28-41% of rNPC patients receiving nasopharyngeal re-irradiation develop NN (4, 6), with approximately 45% of these NN cases involving the internal carotid artery. This progression leads to lethal NN, resulting in massive hemorrhage and even death, significantly impacting patient survival (7). Early prediction of NN risk and individualized interventions before or during treatment are crucial; these interventions include administering prophylactic agents like Endostar (recombinant human endostatin) prior to or early in radiotherapy (8), utilizing precise techniques to minimize high-dose exposure in predicted risk zones, or even opting for alternative comprehensive therapies instead of re-irradiation. However, reliable biomarkers and methods for the accurate prediction of NN in clinical practice remain elusive.

Previous studies primarily used logistic regression to analyze single clinical features of NN risk (9, 10). Yu et al. (10) developed a mathematical model based on multiple clinical features to predict the risk of NN in patients with rNPC receiving IMRT. Nevertheless, the predictive value of models based solely on clinical features is limited, and the underlying pathophysiological mechanisms are largely unknown (11, 12).

Recently, radiomics and dosiomics have gained attention in the field of tumor radiotherapy. Radiomics analysis quantifies image features across different spatial scales, linking individualized physiological and biological information to potential responses to external perturbations such as radiation exposure (13). These analyses reveal associations between various biological features and radiation sensitivity and tolerance. Liu et al. (14) established an NN prediction model for rNPC re-irradiation using multiparametric magnetic resonance imaging (MRI) radiomics and machine learning, which outperformed single clinical factors. Additionally, dosiomics extracts high-dimensional dose spatial features from patient radiation treatment plans, providing richer tissue dose-related information than traditional dose statistics. This approach has been widely applied to analyze radiation dose effects and predict radiation-related responses (15–18).

Deep learning methods offer advanced feature extraction capabilities, leveraging the advantages of radiomics and dosiomics in medical data modeling (19). These models can simultaneously handle multiple clinical tasks and have demonstrated satisfactory results in prognosis prediction (20–22). Although previous studies have applied deep learning to predict normal tissue necrosis after re-irradiation in patients with rNPC (23, 24), there is currently no research combining multi-sequence radiomics features with automatically learned dose-image features for joint modeling. To address this, we collected pre-treatment multi-sequence MRI scans from rNPC patients and extracted handcrafted radiomics features alongside high-dimensional dose image features learned via 3D convolutional neural networks (3D CNNs). Based on these multi-omics features, we developed a neural network prediction model aimed at achieving precise individualized prediction of normal tissue necrosis following re-irradiation, thereby providing valuable guidance for treatment strategy selection and radiotherapy planning.

## Materials and methods

### Patient data

This retrospective study included 117 patients with NPC diagnosed and treated at our center between April 2008 and December 2016. Inclusion criteria were: (1) absence of NN before recurrence; (2) pre-treatment MRI scans with clear T1, T2, and T1C sequences free from artifacts or misalignment; (3) receipt of re-irradiation with intensity-modulated radiotherapy (IMRT); (4) no other malignancies; and (5) adherence to scheduled follow-up every 1–3 months in the first year, every 6 months in the second year, and annually thereafter. The study was approved by the Ethics Committee of Sun Yat-sen University Cancer Center (Number: B2023-103-01) and conducted in accordance with the Declaration of Helsinki, with waived need for written informed consent due to its retrospective nature.

In this study, two datasets were collected, namely dataset A and dataset B cohorts. Dataset A cohorts (n=97) was used as the training set, and dataset B cohorts (n=20) was used as the independent test set. Baseline clinical characteristics showed no significant differences between the two groups (p>0.05). At last follow-up, 32 (33.0%) patients in the training set and 8 (40.0%) in the test set experienced necrosis (NN) (p>0.05). The necrosis-to-non-necrotic sample ratio was approximately 1:2 in the training set and 1:1.5 in the test set (**Table 1**).

**Table 1:** Baseline characteristics in dataset A and dataset B cohorts.

Characteristic	Dataset A cohorts (N=97)	Dataset B cohorts (N=20)	*P*-value^a^
Age, mean (range), years	47 (24–75)	44 (28–62)	0.155
Sex, No. (%)			0.823
Male	75	15	
Female	22	5	
Overall stage^b^, No. (%)			0.208
I	1 (1.0%)	0 (0.0%)	
II	9 (9.3%)	5 (25.0%)	
III	43 (44.3%)	9 (45.0%)	
IV	44 (45.4%)	6 (30.0%)	
Treatment			0.948
No chemotherapy	14 (14.4%)	3 (15.0%)	
Chemotherapy	83 (85.6%)	17 (85.0%)	
Tumor volume (ml), mean	48.9 (1.2–170.2)	39.4 (1.8–156.79)	0.250
rGTV Prescription dose EQD2 (Gy)	63.4 (46.9–71.1)	62.3 (50.0–70.7)	0.177
Number of NN	32 (33.0%)	8 (40.0%)	0.547

a *P* values were calculated using the chi-square test for categorical variables and non-parametric test for continuous variables.

b According to the 8th edition of the International Union against Cancer/American Joint Committee on Cancer (UICC/AJCC) staging manual.

rGTV, recurrent gross tumor volume; NN, Nasopharyngeal necrosis.

### Treatment

All patients included in the study underwent re-irradiation with curative intent using an IMRT planning protocol similar to that previously reported (25). The re-irradiated prescribe ranged from 50–70 Gy, delivered in fractions of 1.80-2.50 Gy per session, five times a week. The average equivalent dose in 2-Gy fractions (EQD2) was 63.4 Gy. Most patients also received platinum-based chemotherapy, including concurrent chemoradiotherapy (CCRT), induction chemotherapy (ICT), and adjuvant chemotherapy (ACT). The ICT or ACT regimens consisted of PF (Cisplatin+5-Fluorouracil), TPF (Cisplatin+5-Fluorouracil+Paclitaxel), or TP (Cisplatin+Paclitaxel) for 2–3 cycles every 3 weeks. CCRT involved cisplatin-based regimens administered at weeks 1, 4, and 7 during radiotherapy, or weekly therapy.

### Diagnosis of NN

NN was diagnosed based on a combination of clinical symptoms, MRI, endoscopic observations, and pathological examinations. The key diagnostic features include the presence of a foul nasal odor, persistent headache, necrotic tissue, skull base osteoradionecrosis visible during endoscopy, discontinuous nasopharyngeal mucosa, or tissue defects observed on MRI, and the presence of a large amount of red-stained substance lacking cellular structure observed by hematoxylin-eosin staining during pathological examination (26). Patients who died of refractory epistaxis and were diagnosed with NN were recorded as having lethal nasopharyngeal necrosis.

### Pre-treatment MRI acquisition and tumor segmentation

Multiparametric MRI scans were conducted for each patient within 2 weeks prior to any antitumor retreatment. Using a combined head-and-neck coil, the scanning range encompassed the suprasellar cistern to the inferior margin of the sternal end of the clavicle. Prior to contrast injection, T1-weighted MR images were obtained in the axial, coronal, and sagittal planes, while T2-weighted MR images were captured in the axial plane. Following intravenous injection of Gd-DTPA at a dose of 0.1 mmol/kg body weight, axial and sagittal contrast-enhanced T1-weighted MR scans were sequentially performed, approximately 40 seconds later. The main acquisition parameters for the scans are listed in (**Supplementary Table 1**).

**Figure 1:**
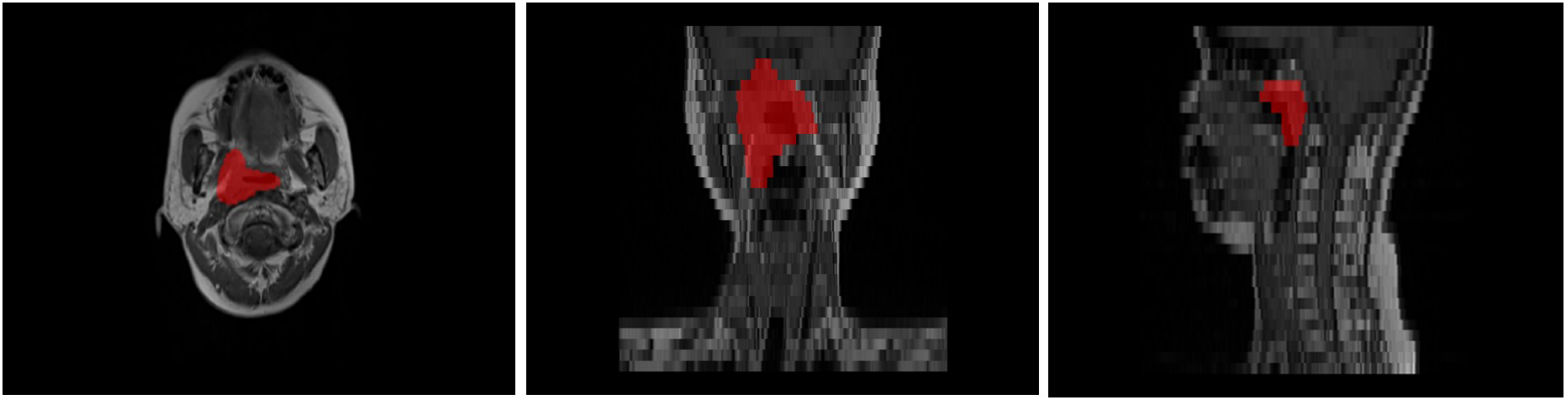
Example of ROI segmentation.

MR images of all patients were imported into MIM software (MIM Vista V7.1.3, MIM Software Inc., Cleveland, OH). Subsequently, the recurrent gross tumor volume (rGTV) was manually delineated and confirmed on each axial MR slice by three experienced radiation oncologists, as shown in **Figure 1**. To ensure inter-observer consistency, the three oncologists independently delineated ROIs and then conducted joint review sessions to resolve any discrepancies and reach a consensus on the final delineations. After delineating the rGTV, the planning target volume (PTVnx) is automatically generated with a 3-mm expansion margin serving as the region of interest (ROI).

### Feature extraction

#### Radiomic feature extraction and selection

*MR image processing and feature extraction:* The PyRadiomics software package (PyRadiomics 3.0.1) was employed to extract PTVnx region features annotated in the T1, T2, and T1C MRI sequences. To mitigate the effect of varying MR image resolutions on the experiments, the image sizes and voxel dimensions were standardized through resampling. Additionally, N4BiasFieldCorrection was utilized to correct bias fields in the MR images (27), thereby reducing the grayscale variations caused by MR scanner discrepancies and other unknown factors.

*Feature selection:* The purpose of feature selection is to remove redundant variables, retain highly correlated features with representative characteristics, enhance the model interpretability and algorithm fitting speed, and avoid overfitting. In this study, the chi-square test was used to preliminarily screen the features in each of the three feature sets. Subsequently, the Least Absolute Shrinkage and Selection Operator algorithm was employed for multivariate regression analysis of omics features (28), selecting highly correlated features predictive of NN. This approach aims to improve the accuracy of the prediction models. Finally, 32 relevant features were selected for training the deep learning model; the specific feature parameters are outlined in (**Supplementary Table 2**).

### Deep learning-based dosiomics feature extraction

**Figure 2:**
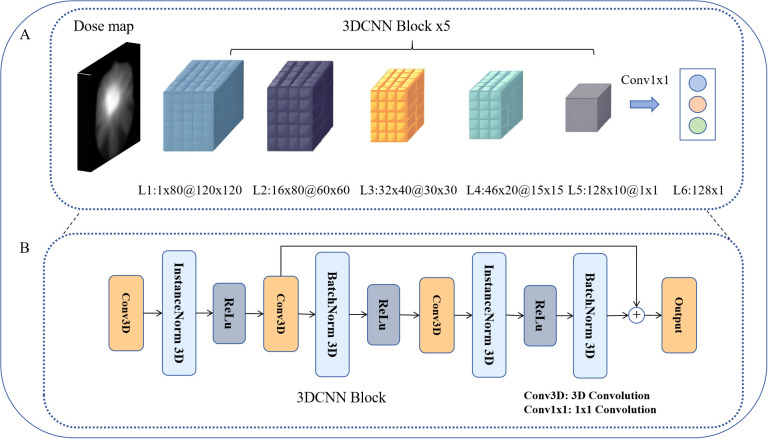
3D CNN architecture. **(A)** 3D CNN architecture for dose–feature extraction. **(B)** 3D CNN block, as the basic module of dose-feature extraction.

We employed the three-dimensional (3D) CNN method for automatic feature extraction directly from raw input dose distribution data (29), as illustrated in **Figure 2**.

This network uses the 3D dose matrix corresponding to the PTVnx, which is cropped from the registered dose map according to the delineated PTVnx region, resulting in an input of 80 dose distribution slices with a resolution of 120 × 120 for each slice. We selected the PTVnx region for analysis as it encompasses the gross tumor volume and its expansion margins, representing the critical area receiving high radiation doses where nasopharyngeal necrosis is most likely to occur. Through 3D convolutional kernels, the network performs convolutional operations on cubes formed by multiple consecutive slices, enabling the feature maps of each convolutional layer to connect to multiple consecutive dose layers (slices). This facilitated the capture of dose distribution feature information across different dose layers. The *j*th feature map of the *i*th layer at the dose position p(x,y,z) is given by the following Equation 1:


(1) $$v_{ij}^{xyz}=relu\left(b_{ij}+\sum_{m}\sum_{p=0}^{P_{i}-1}\sum_{q=0}^{Q_{i}-1}\sum_{r=0}^{R_{i}-1}W_{ijm}^{pqr}V_{\left(i-1\right)m}^{\left(x+p\right)\left(y+q\right)\left(z+r\right)}\right)$$

Where $R_{i}$ , $P_{i},\;\text{and }Q_{i}$ represent the temporal, height, and width dimensions of the three-dimensional convolutional kernel, respectively, and $W_{ijm}^{pqr}$ denotes the value at position (p, q, r) of the connection from the previous layer to the *m*th feature map.

In the network architecture, we utilized 3×3×3 sized 3D convolutional kernels. By progressively increasing the number of channels using 3D CNN blocks, we ultimately obtained a result of 128×1×1 through the output of a 1×1 convolution in layer L6. This task involves fusing dosimetric omics features for classification within a 128-dimensional dose–feature vector space. The 3D CNN block served as the fundamental module constituting the dose–feature extractor (**Figure 2B**). Initially, the input vector undergoes channel expansion via the first layer of 3D convolution. InstanceNorm was applied for spatial normalization, followed by a second 3D convolutional layer for slice-wise downsampling. By employing residual connections, the feature loss is minimized, thereby enhancing the trainability of the model.

### Prediction model building

**Figure 3:**
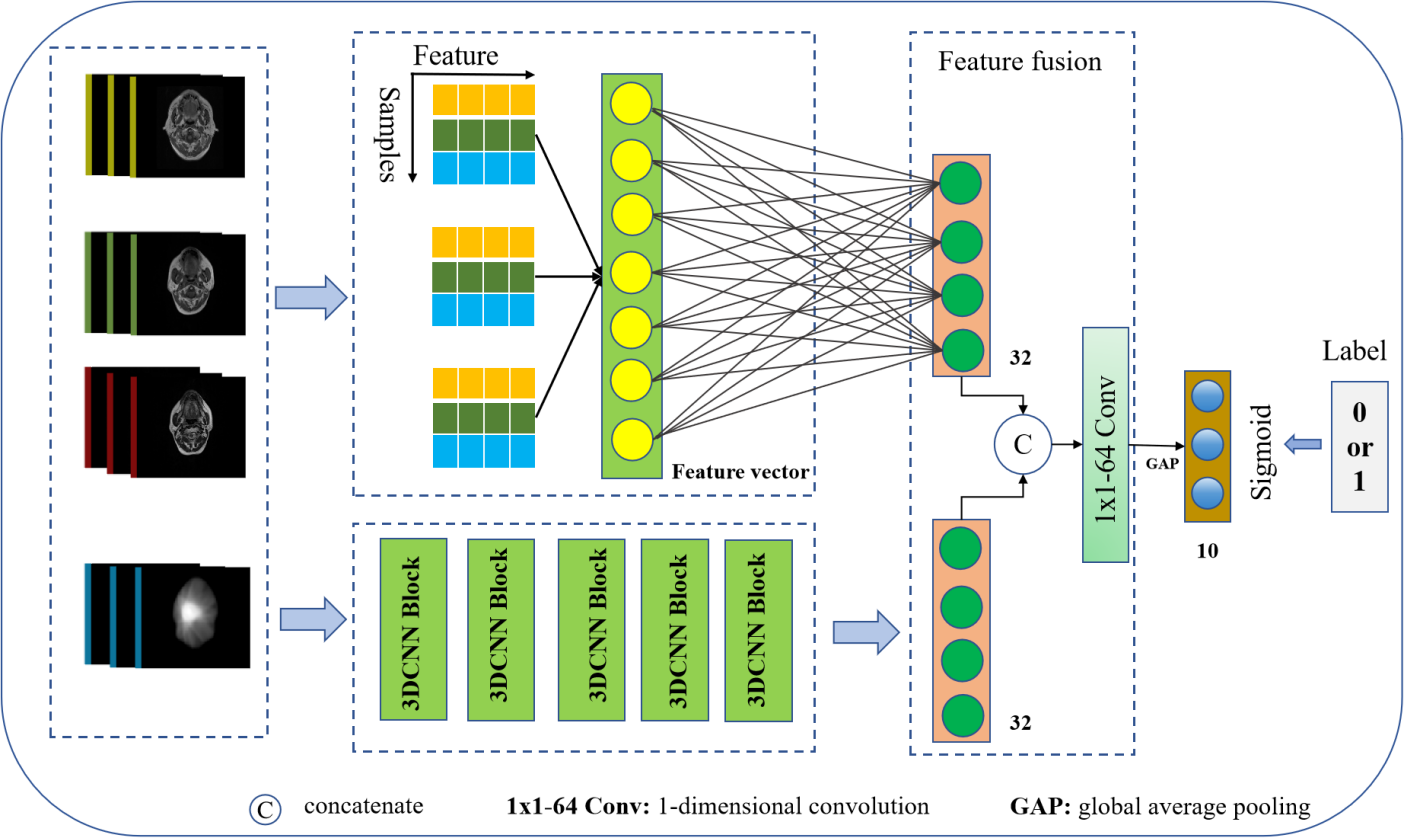
Deep learning model workflow.

We constructed a CNN model architecture to predict NN in rNPC after Re-irradiation by integrating multi-omics features, as illustrated in **Figure 3**. The network takes MR images, annotated target volume, selected imaging omics features, and dose maps as inputs, and outputs the prediction of NN occurrence. It comprises modules for dose–feature extraction, multifeature fusion, and classification.

The dose–feature extractor utilizes a 3D CNN to extract 128-dimensional dosiomics features relevant to the NN using deep learning methods. These features are then projected onto 32 dimensions through a fully connected layer. After manual selection, each MRI imaging omics feature set retained 32 dimensions. These features were concatenated and projected onto 32 dimensions through another fully connected layer for fusion with dose features.

Dosiomics and radiomics features were linearly embedded, stretched into one-dimensional vectors, concatenated, and subjected to a 1×1×64 convolution operation for feature fusion. Subsequently, a one-dimensional global maximum pooling operation is applied, followed by a fully connected layer to obtain the network output.

We aimed to predict the probability of an NN representing a typical binary classification model. Finally, the network employs a sigmoid function to map the output values of the neurons. Given that the last fully connected layer outputs $x_{last}$ , the final network output $x^{pred}$ is determined using the following formula (Equation 2):


(2) $$x^{pred}=sigmoid\left(x_{last}\right)=\left(\frac{1}{1+e^{-x_{last}}}\right)$$

The network output activation function ranged from 0 to 1. Therefore, we established the loss based on the form of the cross-entropy, and the contribution corresponding to $x^{pred}$ is as follows (Equation 3):


(3) $$L=\frac{1}{N}\sum_{i}L_{i}=\frac{1}{N}\sum_{i}-\left[y_{i}.\log\left(x_{i}^{pred}\right)+\left(1-y_{i}\right).\log\left(1-y_{i}^{pred}\right)\right]$$

Here, $L_{i}$ represents the distance between the true label and the predicted value for a single sample, where $y_{i}$ denotes the label of the sample, and $x_{i}^{pred}$ represents the network output. Notably, as the probability of necrosis deviates more from the true label, the penalty for offsetting these values increases, facilitating optimization by the optimizer.

### Establishment of NN prediction models based on radiomics

Training, validation, and testing were conducted separately on three single-sequence MRIs (T1, T1C, and T2) and on the combination of multiple sequences (T1+T1C+T2). For combination, we first concatenated the selected radiomic features from all relevant MRI sequences. To ensure that the input dimension to the neural network remained consistent across all models, we then applied principal component analysis (PCA) to reduce the combined feature vector to the same dimensionality as that of a single sequence. All subsequent operations, network structures, and parameters were kept the same for each model. As a result, four intelligent prediction models for rNPC radiotherapy necrosis based on MRI radiomics were developed: Model_T1_, Model_T1C_, Model_T2_, and Model_T1+T1C+T2_.

### Establishment of NN prediction models based on radiomics and dosiomics

Building upon the prediction models, dosiomic features were incorporated. Training, validation, and testing were conducted on three single-sequence MRIs scans (T1, T1C, and T2) and a multi-sequence combination (T1+T1C+T2). For each sequence, all operations, network structures, and parameters were consistent. To address class imbalance in the training set (necrosis-to-non-necrosis ratio of 1:2), the Synthetic Minority Over-sampling Technique (SMOTE) (30) was employed to balance the classes and improve model robustness, resulting in an effective ratio of approximately 1:1. Four intelligent prediction models integrating MRI radiomics and dosiomics fusion features were obtained: ModelT1+Dose, ModelT1C+Dose, ModelT2+Dose, and ModelT1+T1C+T2+Dose.

The prediction models were implemented using PyTorch, and model training was conducted using two NVIDIA P6000 graphics cards. The Adam optimizer was utilized to optimize the loss function, with parameters β_1_=0.9, β_2_=0.999, learning rate=0.001, and decay=0.00001. The corresponding batch size and number of training epochs were set to 16 and 300, respectively. A grid search strategy was employed for hyperparameter optimization to evaluate different combinations of hyperparameter values.

### Verification of the model

Five-fold cross-validation was utilized for model hyperparameter optimization. We constructed two datasets for comprehensive evaluation: dataset A (n=97) and dataset B (n=20). In dataset A, each fold consisted of a training set (approximately 78 samples) and a validation set (approximately 19 samples), allowing the model to undergo hyperparameter adjustment and validation on 4/5 and 1/5 of dataset A, respectively. After model development and tuning with dataset A, dataset B was used as an independent external test set to further verify the performance of all eight deep learning models across different MRI sequence combinations. The performance of the prediction model was evaluated using AUC (Area Under Curve), ACC (accuracy), and F1-score. AUC is defined as the area under the Receiver Operating Characteristic (ROC) curve, with values ≤1. The ROC curve was plotted based on different binary classification methods (thresholds or decision thresholds), with the true positive rate (sensitivity) as the y-axis and the false positive rate (1-specificity) as the x-axis. An AUC closer to 1.0 indicates a higher predictive ability of the model.

Based on the predicted values matching the true values, four outcomes can be derived: True Positive (TP), predicted values and true values are both positive; False Positive (FP), predicted values are positive while true values are negative; False Negative (FN), predicted values are negative while true values are positive; and True Negative (TN), predicted values and true values are both negative.

Accuracy (ACC) is defined as the number of correctly classified samples divided by the total number of samples (Equation 4):


(4) $$ACC=\frac{TP+TN}{TP+FP+FN+TN}\;$$

ACC approaches 1 as it nears, indicating higher correctness and better predictive capability of the model. The F1-score is defined as the harmonic mean of precision and recall (Equation 5), serving as a metric to gauge the precision of the binary classification models:


(5) $$F1-score=2\times \frac{\text{precision}\times \text{recall}}{\text{precision}+\text{recall}}$$

Where precision=TP/(TP+FP), recall=TP/(TP+FN). The maximum and minimum values were 1 and 0, respectively.

### Statistical analysis

IBM SPSS Statistics (Version 25, SPSS Inc, Chicago, IL) was used to conduct chi-square tests for categorical variables and non-parametric tests for continuous variables. Statistical significance was set at p<0.05.

## Results

Eight deep learning prediction models for rNPC with NN after re-irradiation were first trained and validated using five-fold cross-validation on Dataset A. Subsequently, the models were retrained on the entire dataset A with optimal hyperparameters and externally validated on dataset B. Specifically, we performed a 5-fold cross-validation in each model of the dataset A using the same network architecture and training strategies. The results show that the predictive models based on single-modal MRI radiomics feature have poor performance, with an AUC value of <0.70. The AUC, ACC, and F1-score for Model_T1C_ and Model_T1+T1C+T2_ were 0.67 and 0.68, 0.65 and 0.70, and 0.63 and 0.70, respectively (**Table 2**).

**Table 2:** The evaluation metrics of 8 deep learning models on dataset A.

DL Model	AUC	ACC	F1-Score
Model_T1_	0.66	0.65	0.53
Model_T1C_	0.67	0.70	0.63
Model_T2_	0.67	0.55	0.30
Model_T1+T1C+T2_	0.68	0.70	0.63
Model_T1+Dose_	0.75	0.75	0.70
Model_T1C+Dose_	0.76	0.73	0.78
Model_T2+Dose_	0.78	0.75	0.73
Model_T1+T1C+T2+Dose_	0.86	0.80	0.81

**Figure 4:**
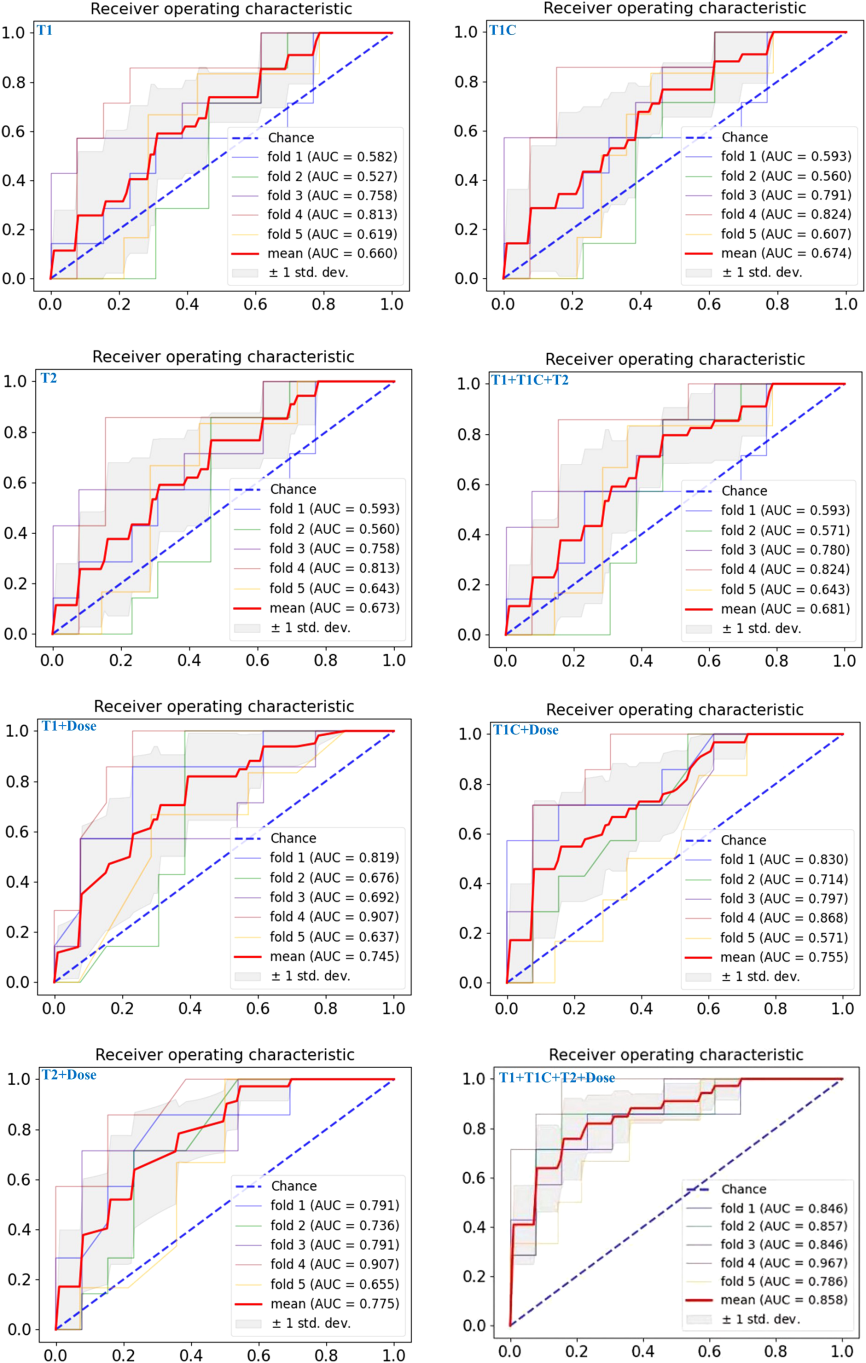
The ROC curves of the eight predictive models on the test set for five-fold cross-validation are shown from left to right, top to bottom, respectively: Model_T1_, Model_T1C_, Model_T2_, Model_T1+T1C+T2_, Model_T1+Dose_, Model_T1C+Dose_, Model_T2+Dose_, Model_T1+T1C+T2+Dose_.

Compared to prediction models based solely on single-modal radiomics features, deep learning intelligent prediction models combining both MRI radiomics and dosiomics features exhibited significantly improved accuracy, whether using a single MR sequence model or multi-sequences MRI model. The AUC, ACC, and F1-score were >0.75, >0.73, and >0.70, respectively. Furthermore, the model (Model_T1+T1C+T2+Dose_) combining radiomics features from multi-sequences MRI (T1+T1C+T2) and dosiomic features demonstrated the best predictive performance, with AUC, ACC, and F1-score values of 0.86, 0.80, and 0.81, respectively (**Table 2**). **Figure 4** showed the AUC results of the five cross-validations.

These performance patterns were maintained in the external validation cohort (**Table 3**), where single-sequence MRI models showed AUCs between 0.64 and 0.66, and the multi-sequence model reached 0.63. The addition of dosiomics features again improved performance across all models, with ModelT1+T1C+T2+Dose achieving the highest metrics: AUC of 0.81, ACC of 0.78, and F1-score of 0.74 in the validation cohort, confirming the consistent performance advantage of the multi-modal integrated approach across different patient populations.

**Table 3:** The evaluation metrics of 8 deep learning models on dataset B.

DL Model	AUC	ACC	F1-Score
Model_T1_	0.65	0.65	0.63
Model_T1C_	0.66	0.65	0.63
Model_T2_	0.64	0.50	0.44
Model_T1+T1C+T2_	0.63	0.70	0.70
Model_T1+Dose_	0.71	0.75	0.74
Model_T1C+Dose_	0.74	0.70	0.70
Model_T2+Dose_	0.72	0.70	0.70
Model_T1+T1C+T2+Dose_	0.81	0.75	0.74

## Discussion

NN is a major and severe complication during re-irradiation treatment for rNPC, significantly affecting patient prognosis. Early and accurate prediction of NN risk is essential for individualized treatment planning and optimization of re-irradiation schedules.

In single-factor analysis, the radiation dose is considered an important risk factor affecting the severity of necrosis. When the cumulative irradiation dose of the gross tumor volume exceeds 141.5 Gy, the incidence of NN significantly increases, making it a critical predictive factor (10). Similarly, the re-irradiation dose is an essential dosimetric factor for NN (31, 32). Dosiomics can reveal the spatial distribution characteristics of radiation doses. When combined with radiomic features, the dose effects caused by tissue heterogeneity can be comprehensively explored. Yang et al. (33) successfully integrated dose radiomics with imaging features to construct a predictive model for radiation-induced temporal lobe injury in NPC radiotherapy, demonstrating superior predictive performance compared with traditional methods. Furthermore, correlations between radiomics features and tissue damage after initial radiotherapy have been found (14), indirectly confirming the potential importance of radiation dose distribution in predicting NN. However, the impact of the re-irradiation dose on the model accuracy is not significant, perhaps because the model incorporated only the prescribed radiation dose without fully incorporating the dosiomics features. In our study, we successfully integrated dosiomics features into predictive models based on radiomics, resulting in significant improvements in prediction accuracy for both single-sequence and multi-sequence models. Notably, the model that combined multi-sequence MRI radiomics with dosiomics demonstrated the most impressive predictive performance, achieving an AUC of 0.86. This surpasses the results reported by Liu et al. (14), further validating the effectiveness of our approach.

The quantitative results in **Tables 2** and **3** provide objective evidence for the advantages of combining multi-sequence MRI radiomics with dosiomics. In the dataset A (**Table 2**), single-sequence MRI models demonstrated limited discriminative ability with AUC values of 0.66-0.67. The combined multi-sequence model (ModelT1+T1C+T2) showed only marginal improvement (AUC=0.68), indicating that simply aggregating different MRI sequences provides minimal additional predictive value.

A marked performance improvement occurred when dosiomics features were incorporated. Single-sequence models combined with dose information achieved substantially higher AUCs (0.75-0.78), while the comprehensive ModelT1+T1C+T2+Dose integrating all data sources reached an AUC of 0.86—representing a 26% improvement over the best single-sequence model and a 10% improvement over the best single-sequence plus dose model. This performance hierarchy was maintained in the dataset B (**Table 3**), where ModelT1+T1C+T2+Dose achieved an AUC of 0.81, significantly outperforming all other configurations.

The external validation data revealed a particularly informative pattern: while the multi-sequence model without dose (ModelT1+T1C+T2, AUC=0.63) performed slightly worse than some single-sequence models (AUC 0.65-0.66), this pattern completely reversed once dosiomics was introduced. This indicates a synergistic relationship between multi-sequence radiomics and dosiomics that enhances predictive capability beyond what either approach achieves independently. The F1-Score data reinforces this conclusion, with ModelT1+T1C+T2+Dose consistently achieving the highest values (0.81 in training, 0.74 in validation), demonstrating that the integrated approach provides more balanced predictions across positive and negative cases.

These results indicate that although each MRI sequence captures different tissue characteristics related to necrosis susceptibility, their predictive potential is maximized when combined with spatial dose distribution information. The substantial AUC increase when dose information is added confirms that tissue susceptibility (captured by radiomics) and radiation exposure patterns (captured by dosiomics) represent complementary dimensions of the necrosis development process. Moreover, we note that Lu et al. (24) proposed a multi-modal deep learning fusion framework based on handcrafted radiomics and dose features on a larger dataset. However, our preliminary attempts to apply their two-stage approach on our cohort produced less satisfactory results, possibly due to dataset characteristics. Therefore, our study focuses on automatic feature learning and assessing the performance benefit of different feature combinations using 3D CNN-based dosiomics, rather than a head-to-head comparison with handcrafted feature-based models.

Precision medicine increasingly relies on integrating multiple data types and artificial intelligence to enhance diagnostic and prognostic accuracy (34). Responding to this trend, our study is the first to combine multi-sequence MRI radiomics characterizing tumor and tissue heterogeneity with high-dimensional dosiomics features extracted via a 3D CNN, enabling precise NN risk prediction after re-irradiation in rNPC patients. This multi-modal approach leverages synergistic information from diverse sources, markedly improving predictive performance and robustness compared to models based on clinical or single-modality data. Our main innovations are: extracting radiomics features from three standard MRI sequences, using deep CNNs for automated spatial dose feature extraction, and achieving personalized risk prediction through multimodal fusion. With an internal validation AUC of 0.86, our method substantially outperforms prior models such as that of Liu et al. (AUC 0.735) (14), underscoring the advantage of deep learning-based multi-omics integration for complex risk prediction.

However, limitations remain. Being a retrospective study from a single center with a limited sample size restricts the model’s generalizability and applicability across different clinical settings. Future multi-center, larger prospective studies are planned to enhance model robustness. Moreover, clinical implementation faces challenges including MRI acquisition standardization, interpretability of deep learning features, model transparency, and integration with physician expertise for individualized radiotherapy adjustments.

In conclusion, this study demonstrates that an AI model based on deep fusion of multi-sequence MRI radiomics and dosiomics features exhibits strong performance and potential in predicting nasopharyngeal necrosis risk after re-irradiation in rNPC patients. It provides robust support for individualized treatment strategy development and re-irradiation optimization, with promising prospects for advancing precision radiotherapy clinical application and improving patient outcomes.

## Data Availability

The datasets presented in this study can be found in online repositories. The names of the repository/repositories and accession number(s) can be found in the article/**Supplementary Material**.
