# Supplementary material for: Deep learning-based multi-omics model to predict nasopharyngeal necrosis of re-irradiation for recurrent nasopharyngeal carcinoma
Source: Front Oncol. 2025 Jul 15;15:1607218. doi: 10.3389/fonc.2025.1607218 (PMC12303931; doi:10.3389/fonc.2025.1607218)
Supplement: Supplementary file 1 [file Table1.docx]

**Supplementary Table 1. MR imaging sequences**

| Sequence | PS (mm) | TE (ms) | FOV (mm) | FA (^o^) | ST (mm) |
| --- | --- | --- | --- | --- | --- |
| T1 | 0.50±0.08 | 7–14 | 200×220-  280×280 | 80-150 | 4.5-6.5 |
| T1C | 0.52±0.08 | 2–22 | 200×200-  280×280 | 75-150 | 4.5-6.5 |
| T2 | 0.51±0.10 | 80–125 | 200×220-  280×280 | 90-150 | 4.5-6.5 |

Abbreviations: PS, pixel spacing; TE, echo time; FOV, field of view; FA, flip angle; ST, slice thickness; T1, T1-weighted sequence; T2, T2-weighted sequence; T1C, T1-weighted contrast-enhanced sequence.

**Supplementary Table 2. The features of MR radiomics**

| Number | Characteristics | Number | Characteristics |
| --- | --- | --- | --- |
| 1 | shape_Flatness | 17 | log-sigma-4-0-mm-3D_firstorder_Mean |
| 2 | firstorder_Skewnessglcm_Idn | 18 | gradient_firstorder_Minimum |
| 3 | shape_Sphericity | 19 | gradient_glcm_Imc2 |
| 4 | ngtdm_Coarseness | 20 | original_glszm_ZoneVariance |
| 5 | glcm_MCC | 21 | 3D_glszm_SizeZoneNonUniformiyNormalized |
| 6 | glrlm_ShortRunLowGrayLevelEmphasis | 22 | gldm_SmallDependenceEmphasis |
| 7 | glszm_GrayLevelNonUniformity | 23 | gradient_firstorder_Skewness |
| 8 | original_glszm_LargeAreaLowGrayLevelEmphasis | 24 | wavelet-LLH_firstorder_Mean |
| 9 | original_glszm_SmallAreaLowGrayLevelEmphasis | 25 | exponential_glrlm_RunEntropy |
| 10 | ngtdm_Busyness | 26 | original_gldm_DependenceNonUniformity |
| 11 | original_firstorder_Minimum | 27 | exponential_glszm_GrayLevelNonUniformity |
| 12 | ngtdm_Strength | 28 | gradient_glcm_Correlation |
| 13 | wavelet-HLH_glszm_ SmallAreaEmphasis | 29 | original_shape_SurfaceArea |
| 14 | wavelet-HHL_glszm_ZoneEntropy | 30 | original_shape_Maximum2DDiameterSlice |
| 15 | wavelet-HHL_glcm_Inverse Variance | 31 | original_shape_Elongation |
| 16 | wavelet-HLH_glcm_Cluster Tendency | 32 | log-sigma-4-0-mm-3D_gldm_DependenceVariance |
